# Ameboma, a rare cause of intestinal obstruction in bariatric surgery patients: a case report

**DOI:** 10.1093/jscr/rjae759

**Published:** 2024-12-11

**Authors:** Mauricio Palacios, Alex Guachilema R, Andrea Lisintuña, Julio Yepez, Sergio Verboonen

**Affiliations:** Cirugía General, Hospital Metropolitano, Av. Mariana de Jesus s/n, Quito 170521, Ecuador; Cirugía General, Hospital Metropolitano, Av. Mariana de Jesus s/n, Quito 170521, Ecuador; Cirugía General, Hospital Metropolitano, Av. Mariana de Jesus s/n, Quito 170521, Ecuador; Médico Internista, Hospital Metropolitano, Quito 170521, Ecuador; Bariatric Surgery, Obesity Goodbye Center, Tijuana 22000, Mexico

**Keywords:** ameboma, abdominal mass, right hemicolectomy, colon cancer, intestinal obstruction

## Abstract

Ameboma is a rare complication of invasive amebiases caused by the intestinal protozoan *Entamoeba histolytica*. The disease is characterized by tumorous, exophytic, inflammatory masses in the lower-right quadrant of the abdomen. Symptoms are nonspecific but may include intestinal obstruction and gastrointestinal bleeding. Although computed tomography and ultrasound can identify colonic masses, biopsy is the most accurate diagnostic method for ameboma. Differential diagnosis includes Crohn’s disease and colon carcinoma. Treatment involves killing the trophozoites in most cases and surgical intervention in patients with intestinal obstruction or perforation, phlegmon, or toxic megacolon. This study reports a case of intestinal obstruction caused by ameboma within the ascending colon of a bariatric surgery patient.

## Introduction

Ameboma is characterized by granulomatous lesions in the lower-right quadrant of the abdomen secondary to invasive amebiasis caused by *Entamoeba histolytica* [1, 2]. The condition usually presents as a segmental and concentric lesion in the gastrointestinal mucosa and may cause luminal narrowing that results in intestinal obstruction and gastrointestinal bleeding [1, 3]. *E. histolytica* infections occur when cysts are ingested through contaminated food or water. Trophozoites cause invasive amebiasis [4]. The diagnosis is difficult because the disease is rare and is made after excluding other causes. Ameboma accounts for 1.5% of all cases of invasive amebiasis [3].

## Case presentation

A 36-year-old man with a history of type 2 diabetes, hypertension, and obesity had undergone Roux-en-Y gastric bypass 10 months prior and visited a health clinic with a 15-day history of diffuse abdominal pain (visual analog scale of 9 to 10), abdominal distension, and vomiting due to intestinal obstruction. Laparoscopy identified a mass in the ascending colon, and loop ileostomy was performed. Abdominal pain and distension persisted postoperatively. The patient visited our clinic, and a physical examination indicated abdominal distension with pain on palpation, gurgling sounds, temporary ileostomy, and peritoneal inflammation.

**Figure 1:**
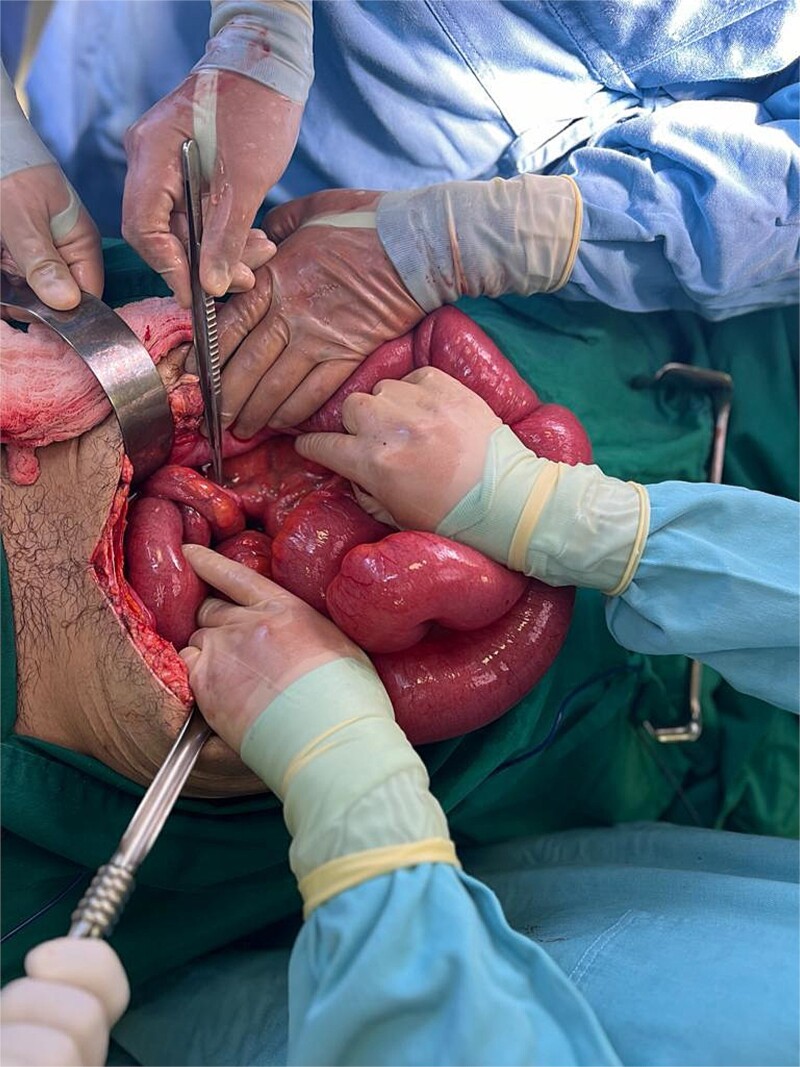
Parastomal hernia and small bowel obstruction after loop ileostomy.

**Figure 2:**
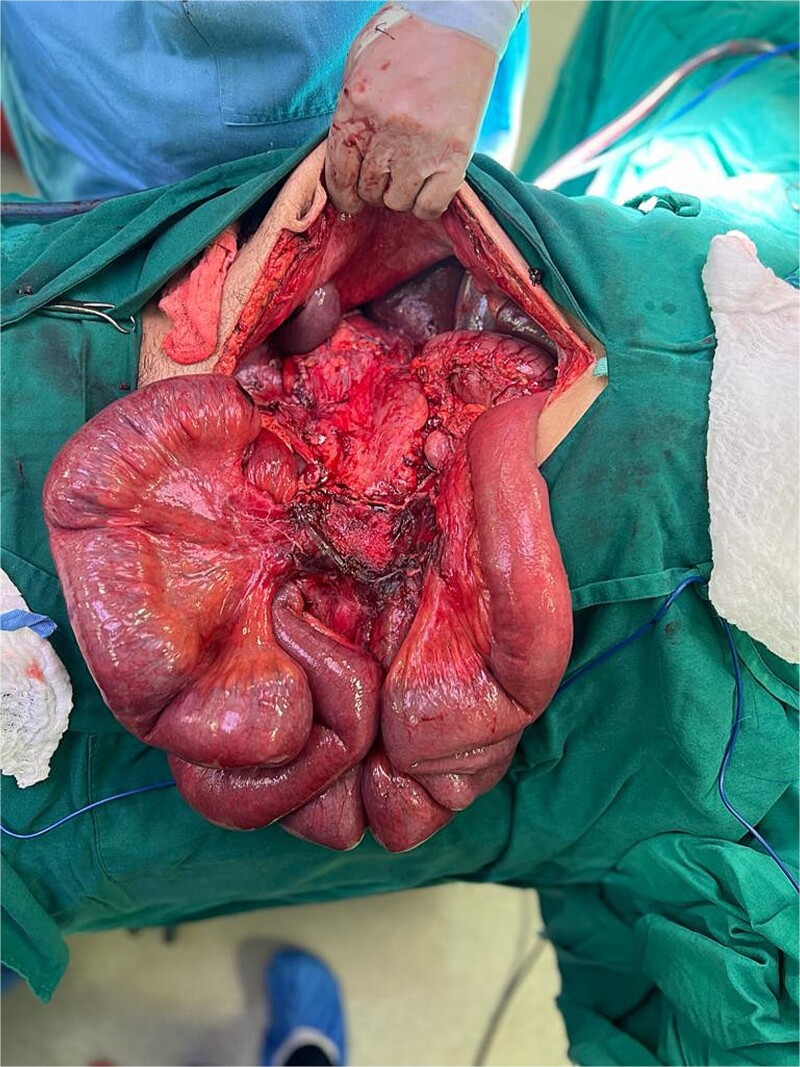
Nodular mass (~20 × 15 cm) in the mesentery of the ascending colon.

**Figure 3:**
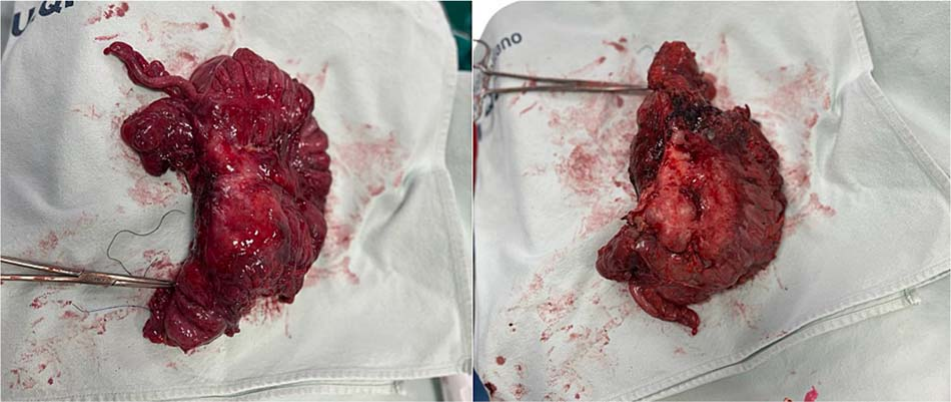
Biopsy sent to pathology.

The results of laboratory tests showed leukocytosis and elevated C-reactive protein, and surgical reintervention was considered. Exploratory laparotomy showed gastrointestinal and enteroenteric anastomoses, parastomal hernia, and small bowel obstruction after loop ileostomy (Fig. 1), and a lobulated, hard, fixed nodular mass (~20 × 15 cm) in the mesentery of the ascending colon with intra-abdominal adhesions (grades 3 and 4 according to Zühlke’s criteria) located in the duodenum, transverse colon, and mesenteric vessels (Fig. 2). We performed the surgical resection of adhesions and granulomatous lesions, right hemicolectomy, and isoperistaltic side-to-side anastomosis of the transverse ileus (Fig. 3).

The patient remained in the intensive care unit with a nasogastric tube for 2 days postoperatively without complications. The tube was removed on the same day, and a soft diet was started. He was discharged without complications. The pathology result was a lesion in the mesentery of the ascending colon, a chronic inflammatory lesion secondary to intestinal perforation, and the presence of *E. histolytica* trophozoites and actinomyces.

## Discussion

Ameboma is a rare complication of amebiases caused by *E. histolytica* [1]. Trophozoites cause invasive amebiases and colonic mucosal lesions, potentially leading to intestinal perforation or obstruction. This study is the first to report a case of an ameboma within the ascending colon. Our patient had an obstructive mass in the cecum and ascending colon, intestinal perforation, and *E. histolytica* trophozoites in the colon.

Amebiasis is the third most prevalent parasitic disease worldwide, and 2970 cases have been reported in the United States, primarily in Latin American immigrants. The mortality rate from amebiasis complications is 0.1% to 0.25% [2, 3]. Patients with chronic amebiasis may develop ulcerative, exophytic, and inflammatory lesions mimicking colonic carcinoma. Amebic infections may be asymptomatic or cause colitis, abscesses, and colonic perforation [2]. *E. histolytica* trophozoites migrate from the colon to the liver via the portal venous system and cause invasive amebiases [2]. Amebomas result from the development of granular tissue in the cecum, cecal appendix, recto-sigmoid junction, hepatic flexure, and transverse colon [2, 3]. The main complications of ameboma are intestinal perforation or obstruction, intussusception, anorectal fistula, and appendicitis [5].

The diagnosis of ameboma is difficult because the condition mimics colon carcinoma. Moreover, ultrasound and contrast-enhanced computed tomography do not detect invasive amebiasis because these methods cannot distinguish between intestinal lesions and cancer. Thus, fluorescence-guided laparoscopic colorectal surgery using indocyanine green fluorescence imaging is required.

Differential diagnosis should include Crohn’s disease, abscesses, colon carcinoma or diverticulitis (in older adults), non-Hodgkin lymphoma, tuberculosis, fungal infections, AIDS-associated lymphoma, and Kaposi sarcoma. Differential diagnosis in bariatric surgery patients includes intestinal obstruction due to Roux-en-Y gastric bypass. The most accurate diagnostic method for ameboma is biopsy, which is performed in 60% of cases. A biopsy should be obtained from the base of the lesion and should detect trophozoites. However, biopsies are negative in approximately one-third of cases because of inadequate sampling or the non-identification of the pathogen by the pathologist [4].

Treatment involves killing the trophozoites in most cases and surgical resection in patients with intestinal obstruction or perforation, phlegmon, or toxic megacolon [6, 7].

Amoeboma is a rare entity, and its complications include perforation and intestinal obstruction, as in our case, where the symptoms were intestinal obstruction and the intervention identified an obstructive mass of the colon located in the cecum and ascending colon involving the mesocolon with a histopathology report of plastron with perforation into the mesocolon with the presence of amoebae.

As described in the literature, it can be difficult to diagnose and can be confused with malignant neoplastic lesions. In the context of a post-bariatric surgery patient, the differential diagnosis will also be obstructive causes derived from the Y gastric bypass.

The case of an accidental amoeboma to the mesocolon is not reported in the literature.

## References

[1] Wang S-Y , Shih S-C, Wang T-E, et al. Ameboma mimicking submucosal tumor of the colon in an elderly. Int J Gerontol 2011;5:126–8.

[2] Luis MA , Carreño M, Ruiz B, et al. Synchronus ameboma in Vater ampulla and colon, first case reported in literature. MOJ Clin Med Case Rep 2016;5:00117. 10.15406/mojcr.2016.05.117.

[3] Hardin RE , Ferzli GS, Zenilman ME, et al. Invasive amebiasis and ameboma formation presenting as a rectal mass: an uncommon case of malignant masquerade at a western medical center. World J Gastroenterol 2007;13:5659–61. 10.3748/wjg.v13.i42.5659. 17948943 PMC4172748

[4] Almalki M , Yaseen W. Cecal ameboma mimicking obstructing colonic carcinoma. J Surg Case Rep 2018;2018:rjy124. 10.1093/jscr/rjy124. 29942473 PMC6007312

[5] Subramaniam SRV , Perumal SK, Rajendran K, et al. An interesting presentation of Ameboma – a case report and review of literature. J Gastroenterol Pancreatol Liver Disord 2018;6:1–4. 10.15226/2374-815X/6/4/00113

[6] Omwansa P , Nyatsambo C, Ngwisanyi W, et al. A case report of colonic Ameboma mimicking colon cancer in an immunocompromised patient. Int J Surg Case Rep 2023;110:108768. 10.1016/j.ijscr.2023.108768. 37657387 PMC10510079

[7] Lin CC , Kao KY. Ameboma: a colon carcinoma-like lesion in a colonoscopy finding. Case Rep Gastroenterol 2013;7:438–41. 10.1159/000355880. 24403882 PMC3884178

